# Complete deltoid resection in early childhood without muscle transfer results in normal shoulder function at long-term follow-up: a case report

**DOI:** 10.1186/s13256-016-1132-z

**Published:** 2017-01-14

**Authors:** Annie Arteau, Franziska Seeli, Bruno Fuchs

**Affiliations:** 1 Hôtel Dieu de Québec, 11 Côte du Palais, Quebec, G1R 2J6 Canada; 2 Sarkomzentrum UZH, University of Zurich, Forchstrasse 340, CH-8008 Zurich, Switzerland

**Keywords:** Deltoid resection, Shoulder function, Pediatric

## Abstract

**Background:**

Musculoskeletal tumors involving the deltoid muscle and necessitating its complete resection are rare. The function after complete deltoid resection is reported to be limited, and several authors consider muscle transfer to improve shoulder motion. However, it still remains unclear whether such transfer adds function. To the best of our knowledge, all reports on complete deltoid resection refer to adult patients, and it is unknown what function results after deltoid resection in childhood. The remaining muscles may have the potential to compensate for the loss of deltoid function.

**Case presentation:**

Here we report the case of a 5-year-old white boy with complete (isolated) deltoid muscle resection in infancy for a large aggressive soft tissue tumor. No reconstructive procedure or muscle transfer was performed at the time of index surgery. Pathology revealed an angiomatoid fibrous histiocytoma. His postoperative course was uneventful. At 11 years of follow-up, he remained disease-free and had excellent shoulder function, including normal range of motion.

**Conclusions:**

This report implies that major muscles such as the deltoid can be resected in a child without compromising long-term function. Therefore, a muscle transfer at index surgery is probably not necessary.

## Background

Most of the knowledge related to deltoid dysfunction stems from patients with non-neoplastic pathologies such as brachial plexus injuries, axillary nerve palsy secondary to shoulder luxation, iatrogenic nerve or muscle lesions, or after poliomyelitis [1, 2]. Loss of anterior deltoid function had been historically associated with impairment of glenohumeral function and chronic pain [1–5]. When associated with a rotator cuff tear, impairment is higher, nerve reconstruction or muscle transfer success is lower [1, 3, 4, 6].

Muscle or nerve transfer or graft for deltoid insufficiencies of any cause is associated [1, 3–6] with variable success rates. Only a few authors reported a complete range of motion with complete deltoid palsy without any reconstructive procedure [6]; they suggested that sufficient function could be obtained to allow full duty work.

Complete resection of the deltoid muscle for musculoskeletal tumors is rarely performed [7, 8]. In the tumor literature, where the loss of the deltoid muscle can be regarded as an isolated lesion with a normal rotator cuff, it is thought that partial resection of the deltoid muscle may result in normal shoulder function [8, 9], whereas complete resection compromises its function. Because of the purported limited function, consideration is often given to the addition of a latissimus dorsi, a trapezius, or a pectoralis major transfer to potentially improve the functional outcome [10, 11].

Therefore, there is continuing debate on whether a muscle transfer may improve shoulder function after complete deltoid resection. Some authors recommend muscle transfer to improve shoulder motion or strength, whereas others recommend muscle transfer solely for tissue coverage because they believe it adds little to the improvement of shoulder function. Of interest, all this literature reports on adult patients and it is unclear whether this also applies to children, who may have a greater adaptability and potential compensation for their shoulder muscle function.

## Case presentation

**Fig. 1:**
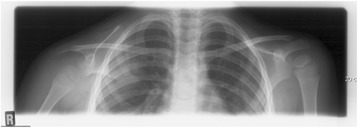
At initial presentation, the radiographs showed an inferior subluxation of the right shoulder

**Fig. 2:**
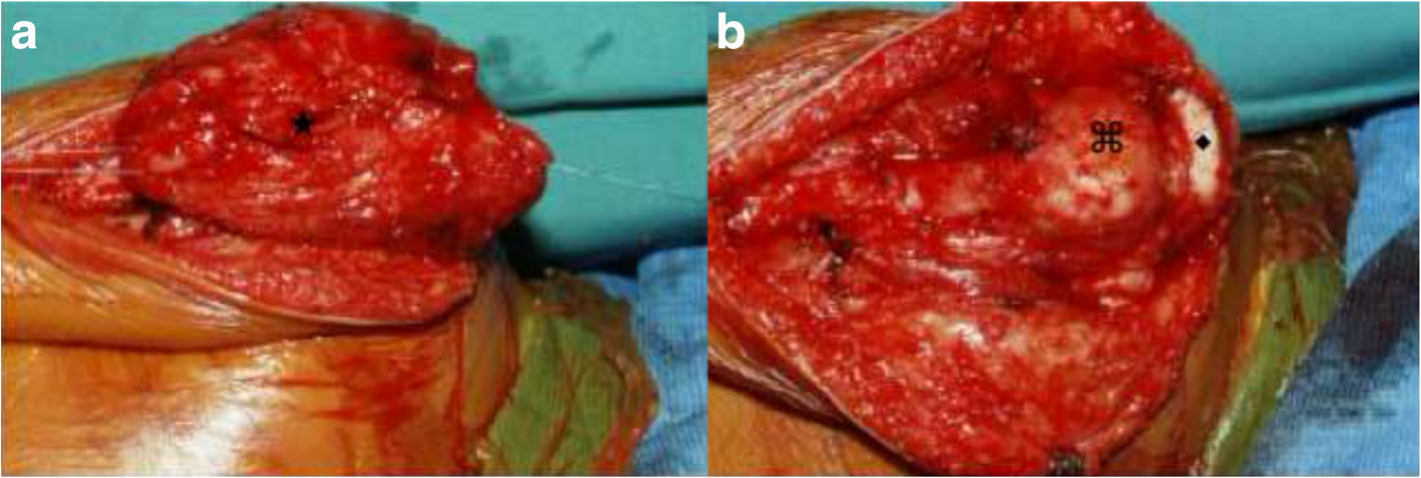
Intraoperative pictures showing infiltration of the muscle and subcutis. **a** The tumor within the deltoid muscle (★). After deltoid muscle resection, the humeral head (⌘) and the acromion (♦) can be seen (**b**)

**Fig. 3:**
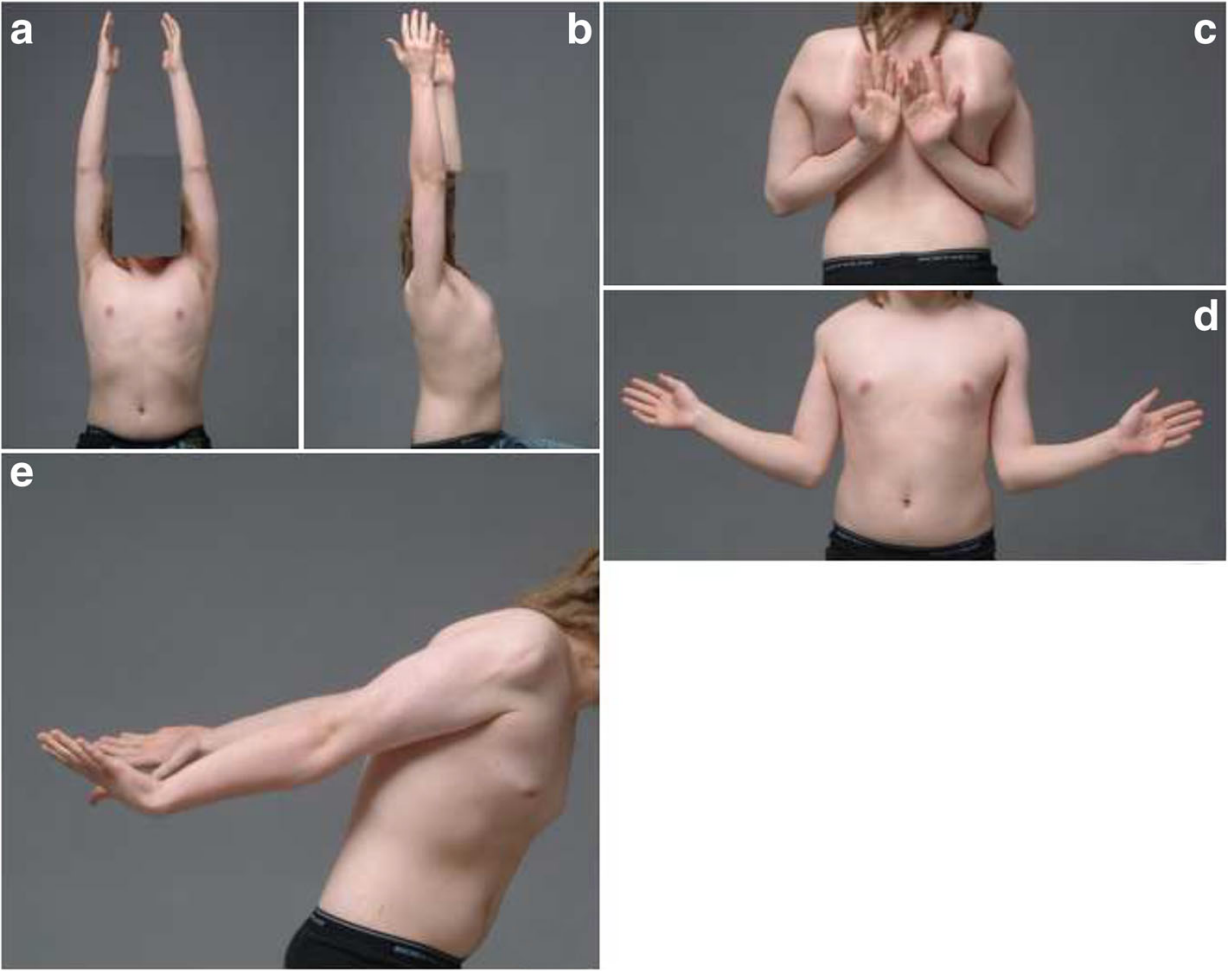
Normal range of motion 9 years after surgery. Our patient is now 16-years old. Abduction in the scapular plane (Fig. 3a), forward elevation (Fig. 3b), internal rotation (Fig. 3c), external rotation (Fig. 3d), and extension are normal (Fig. 3e)

A 5-year-old white boy presented with a 1-year history of right shoulder pain and asymmetrical right scapula. His shoulder range of motion was preserved as well as function. Amyotrophy was initially suspected but excluded based on normal neurologic investigation. There was no significant past medical or family history. Imaging revealed a persistent subluxation of the humeral head of unclear genesis (Fig. 1). Subsequent investigations by computed tomography (CT) scan and MRI showed a 5×3.5×2.5 cm soft tissue mass with an aggressive appearance within his deltoid muscle but without involvement of his brachial plexus. An initial biopsy failed to determine the exact nature of the lesion but revealed an aggressive biology. Based on the clinical and aggressive appearance of the lesion on imaging, the remaining diagnosis was a malignancy. Consequently, a complete deltoid resection was performed on this child because of the infiltrative pattern of the tumor through the entire muscle (Fig. 2). Surgery included a straight lateral, vertical incision from his acromion to the deltoid insertion (Fig. 3e). The posterior border of his deltoid muscle was prepared and the axillary neurovascular bundle identified. Muscular detachments from the deltoid insertion and then his acromion followed, before his entire muscle was mobilized together with the tumor. No reconstructive procedure was performed at the time of index procedure or later.

**Fig. 4:**
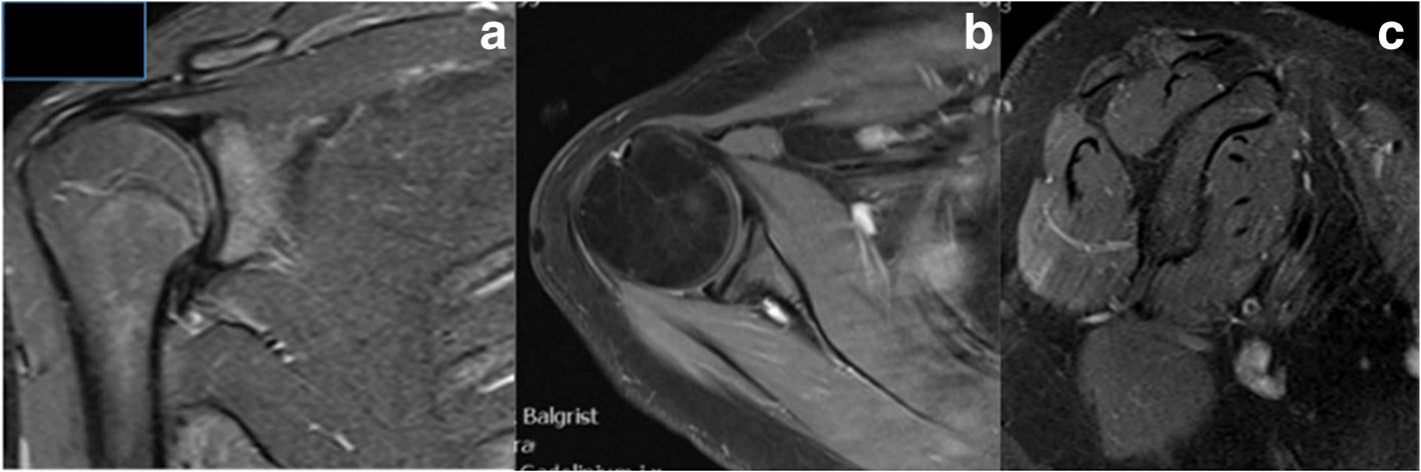
Magnetic resonance imaging (Fig. 4a frontal plane; Fig. 4b axial plane; Fig. 4c parasagittal plane) of the patient’s shoulder at 11 years of follow-up shows normal rotator cuff muscles without atrophy and fatty streaks, but the absence of the deltoid muscle

Histological analysis of the tumor revealed an angiomatoid fibrous histiocytoma. At the time of diagnosis, this lesion was known as angiomatoid malignant fibrous histiocytoma because initially its metastatic potential was considered much higher than it is now. Angiomatoid fibrous histiocytoma is a low grade lesion with a 1% metastatic rate. Complete surgical resection is usually curative. No adjuvant therapy was performed. His postoperative course was uneventful. Over the first few weeks, physical therapy (PT) assisted in mobilizing his shoulder joint. Six months postoperatively, the child demonstrated near complete range of motion of his right shoulder. Over the years, the growing child participated in normal physical activities and maintained a symmetric range of motion. Ten years after the complete deltoid resection, he is free of disease and his shoulder function remains excellent. When compared to his left side, the range of motion of his right shoulder is symmetrical and normal (Fig. 3). Normal rotator cuff muscles and good centralization of the humeral head were observed without degenerative changes on a recent MRI at 11 years of follow-up (Fig. 4). His Musculoskeletal Tumor Society Score (MSTS; a score widely used in orthopedic oncology; it addresses pain, function, emotions, hand positioning, manual dexterity, and lifting ability) for upper extremity is excellent (93.3%), his Constant Shoulder Score (a score widely used in shoulder surgery; it addresses pain, activity of daily living, arm positioning, range of motion, and abduction power) is 75.8% on his right side and 80% on his left side. Patient self-assessment of his shoulder function (subjective shoulder value) is excellent (95%) with only slight weakness noted when heavy objects are carried. Despite excellent function reported, a slight difference to the opposite side is observed. Objectively, his shoulder strength in forward elevation is 54.6 kg, internal rotation is 20.8 kg, and external rotation is 15.8 kg compared to 76kg, 23.4 kg, and 24.1 kg respectively for his contralateral shoulder. MRI revealed an intact rotator cuff without atrophy of his supraspinatus, infraspinatus, and subscapularis muscles (Fig. 4).

## Discussion

We can only speculate about the reasons why there is normal shoulder function despite the complete removal of the deltoid muscle. In the normal shoulder, a combination of muscles generates a complex movement: anterior deltoid, supraspinatus, coracobrachialis, long head of biceps brachii, and clavicular portion of pectoralis major act together to provide power and movement in forward flexion. Abduction in the scapular plane is generated by the middle portion of the deltoid, supraspinatus, and a coupled force between subscapularis and infraspinatus [12, 13]. A selective bloc of the suprascapular or axillary nerve showed that the deltoid muscle provides 50% of the power for forward elevation and abduction in the plane of the scapula. Supraspinatus and infraspinatus generate almost 50% of the torque; infraspinatus acts mostly above 90 degrees of abduction. Electromyographic studies [14] showed a silent signal before 90 degrees of abduction. In the absence of deltoid and rotator cuff muscles, other muscles cannot generate enough torque to overcome gravity but act as a movement generator [12, 13]. Of interest, our patient showed excellent strength in abduction in the plane of the scapula without deltoid muscle. In addition, an MRI of his shoulder at 11-years follow-up showed normal shoulder muscles, without intraarticular degenerative changes. Although up to 20% of the patients with a complete deltoid dysfunction may achieve full (passive) range of shoulder motion [6, 15], our patient also had very good strength. Altogether, this implies that the other shoulder muscles can compensate for the loss of deltoid function after its complete removal in early childhood. Isolated deltoid resection is a rare procedure, even more in the pediatric population, and consequently it is difficult to make recommendations regarding the need for reconstruction or not based on this specific case. On the other hand, the excellent long-term follow-up of this patient with an annual physical examination and medical photography have showed that excellent function and strength remain over years without reconstruction.

## Conclusions

To the best of our knowledge, this is the first report of complete deltoid resection without muscle transfer in a child, resulting in a full range of motion, excellent strength, and excellent function of the shoulder at long-term follow-up. The remaining shoulder muscles have the potential to compensate for the loss of deltoid function in very young children thereby avoiding the need for complex procedures like pedicled latissimus muscle flap or trapezius transfer unless needed for soft tissue coverage.
